# Lessons Learned from POCUS Instruction in Undergraduate Medicine During the COVID-19 Pandemic

**DOI:** 10.24908/pocus.v8i1.16410

**Published:** 2023-04-26

**Authors:** Sherwin Wong, Salwa Nihal, Danny Yu Jia Ke, Emma Neary, Luke Wu, Edwin Ocran, Michael Cenkowski, Nicholas Grubic, Stephen C Pang, Amer M Johri

**Affiliations:** 1 Kingston Health Sciences Centre Kingston, ON Canada; 2 Queen's University Kingston, ON Canada

**Keywords:** POCUS, virtual, education, flipped classroom

## Abstract

Point of care Ultrasound (POCUS) has been adopted into clinical practice across many fields of medicine. Undergraduate medical education programs have recognized the need to incorporate POCUS training into their curricula, traditionally done in small groups with in-person sessions. This method is resource intensive and requires sufficient equipment and expertise. These requirements are often cited as barriers for implementation. During the Coronavirus Disease 2019 (COVID-19) pandemic, POCUS education was required to adapt to physical distancing regulations, giving rise to novel teaching methods for POCUS. This article outlines the implementation of a POCUS teaching session before and during the pandemic. It describes how these innovations can scale POCUS teaching and overcome barriers moving forward. A flipped classroom model was implemented for all learners. Learners were given an introductory POCUS module before the scheduled in-person or virtual teaching session. Sixty-nine learners participated in conventional in-person teaching, while twenty-two learners participated in virtual teaching following the pandemic-related restrictions. Learners completed a written test before and following the teaching. In-person learners were assessed using an objective structured assessment of ultrasound skills (OSAUS) pre- and post-learning sessions. A follow-up survey was conducted three years after the teaching sessions were completed. Both in-person and virtual groups demonstrated statistically significant improvement in knowledge scores (p <0.0001). Both groups had similar post-test learning scores (74.2 ± 13.6% vs. 71.8 ± 14.5 %, respectively). On follow-up questionnaires, respondents indicate that they found our online and in-person modes of teaching helpful during their residency. POCUS education continues to face a variety of barriers, including limitations in infrastructure and expertise. This study describes an adapted POCUS teaching model that is scalable, uses minimal infrastructure and retains the interactivity of conventional small-group POCUS teaching. This program can serve as a blueprint for other institutions offering POCUS teaching, especially when conventional teaching methods are limited.

## Introduction

In response to the growing demand for bedside ultrasound skills, medical schools in Canada and internationally have attempted to integrate point of care ultrasound (POCUS) into their curriculum 1, 2. This is traditionally done in small groups with in-person sessions. However, this method is resource-intensive and requires sufficient equipment and expertise. These requirements are often cited as barriers to implementing POCUS curricula, along with curricular time constraints and lack of faculty support 3, 4. Conventional POCUS teaching requires instructors, learners, and a volunteer to be within proximity. The COVID-19 pandemic brought about widespread physical distancing recommendations, forcing educators to adapt the teaching of POCUS and other clinical skills. We describe a formal evaluation and adaptation of one of the first virtual POCUS teaching sessions integrated into a medical school curriculum during COVID-19.

## Study Design

This study included second-year medical students from Queen’s University, Canada, recruited from January to March 2020. A flipped classroom model was implemented for all learners. Learners were provided with an introductory POCUS module before the in-person or virtual teaching schedule. The students were prospectively recruited based on the implementation of physical distancing restrictions at the time: conventional in-person (n = 69) and virtual (n = 22) teaching. Students in the in-person group rotated through an afternoon session with an average of 10 students at each session with three instructors. This study was approved by the Queen's University Health Sciences and Affiliated Teaching Hospitals Research Ethics Board (HSREB #: DBMS-071-17). Participants were provided with a session outline and consent form before arrival at the session and written consent was obtained for the use of the anonymized data.

### POCUS Pre-Session Resource

Students were provided with a custom-made online POCUS module prior to both virtual and in-person sessions. The research group developed the module and integrated feedback from expert POCUS users within the Queen’s community. The module’s primary objective was to provide foundational knowledge and briefly introduce image acquisition and interpretation of normal findings. The module discussed the following: POCUS limitations, applications, basic physics, knobology, image acquisition, and image interpretation for three POCUS views: cardiac parasternal long axis (PLAX), anterior lung and pleura, and right upper quadrant (RUQ) abdominal POCUS examination for free fluid. 

### POCUS In-Person Teaching

The in-person session was divided into three phases: i) Pre-test, ii) Hands-on learning, and iii) Post-test. **Pre-Test and Post-Test:** The pre- and post-tests consisted of 6-minute stations in which students rotated between three written quiz stations and one practical station (Observed Structured Assessment of Ultrasound Skills (OSAUS)). The written stations had five multiple-choice questions each, assessing foundational knowledge covered in the POCUS module. 

The OSAUS station allowed for the assessment of the students’ baseline ability to apply the foundational POCUS knowledge. Students were provided with a POCUS device and asked to generate and identify normal findings on the three POCUS views covered in the module: cardiac PLAX, anterior lung and pleura, and RUQ abdominal POCUS. Image acquisition was formally observed and marked using a checklist (Supplementary Item S1). Standardized patients were used for the exam. Researchers used a standardized assessment form, a modified version of a previously published OSAUS tool 5, 6. The modified assessment tool underwent several iterations and was checked for face validity amongst our research group. 

#### Hands-on learning: 

The hands-on learning session consisted of three 25-minute stations where instructors provided small group instruction (3:1 or 4:1 student-to-instructor ratio) on the three POCUS views, reinforcing concepts taught in the electronic modules and developing practical skills for image acquisition and interpretation. Each group of students rotated through the PLAX, anterior lung/pleura, and RUQ stations during the 75-minute period. The priority of these sessions was to ensure that students maximized the time holding the probe directly. 

### Virtual Session

**Figure 1:**
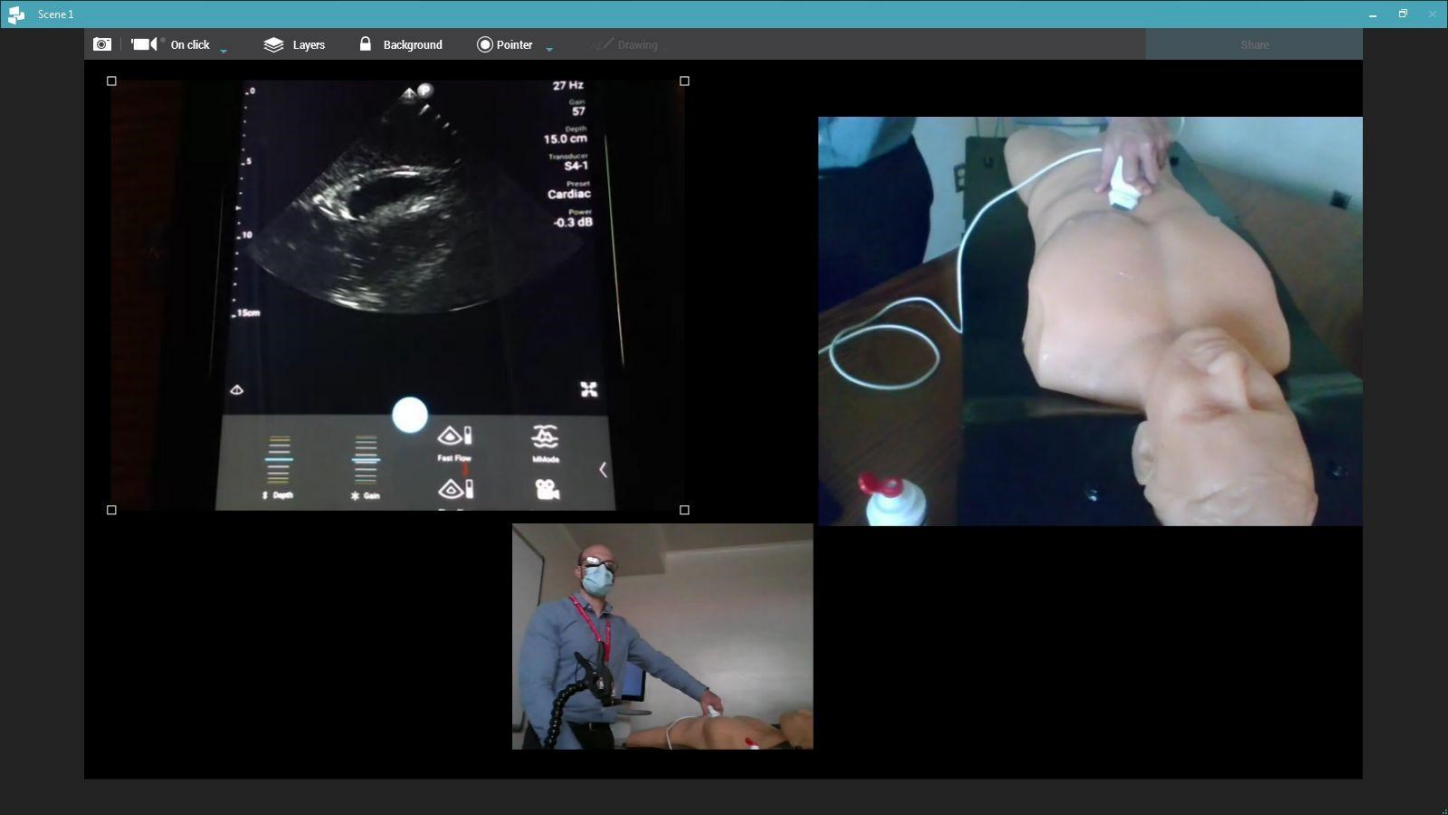
Demonstration of point of care ultrasound device and tablet using a cardiac phantom on a virtual learning platform. Equipment details are outlined in Supplementary Item S2 .

We designed a 90-minute interactive virtual session to deliver POCUS teaching. For this session, learners joined a video conference where they could simultaneously see a screencast of a handheld POCUS device screen and probe placement on surface anatomy via a separate camera feed (Figure 1). During the session, learners could interact with the instructions and visualize how probe movements changed the POCUS image. Further equipment and setup details are in the supplementary material (Supplementary Item S2).

There were two components: i) a brief didactic session that reviewed the foundational knowledge and ii) a live virtual demonstration that allowed for interaction and feedback. The live demonstration included scanning a phantom and an instructor self-scanning. The same written questions were delivered as a pre-test and post-test in the live and virtual sessions. In contrast to the in-person group, there was no assessment of scanning skills in the virtual teaching group. 

### Follow-up survey

Three years after the completion of the teaching sessions, an anonymous survey was distributed to all study participants. The questionnaire focused on the impact of POCUS on their residency training. Study questions included medical specialty, institution, use of POCUS in their clinical work, and reflection on their undergraduate POCUS training. Surveys were sent out via email, and respondents were provided with a $15 gift card for their participation in the survey. Two reminder emails were sent at two-week intervals. 

### Data Collection and Analysis

Written test scores were collected online for both in-person and virtual groups. For the in-person group, OSAUS assessments were completed by a single evaluator at each station during the pre- and post-test phases of the study. For consistency, the same evaluator assessed the same students pre- and post-test in most assessments. Pre- and post-test scores were compared for both written and each component of the OSAUS. Written scores were compared between in-person and virtually taught groups. Mean scores, mean improvement and standard deviations were calculated. Paired Student’s t-test and Wilcoxon signed-rank test were performed. P values <0.05 were deemed statistically significant. Figures were generated using Prism GraphPad Version 8 (San Diego, CA, USA). Survey data were reported with standard descriptive statistics using frequency and percentage distributions. 

## Results

Seven in-person sessions were conducted with a total of sixty-nine participants. Twenty-two learners participated in the virtual teaching session. 

### Written Test Results: 

**Figure 2:**
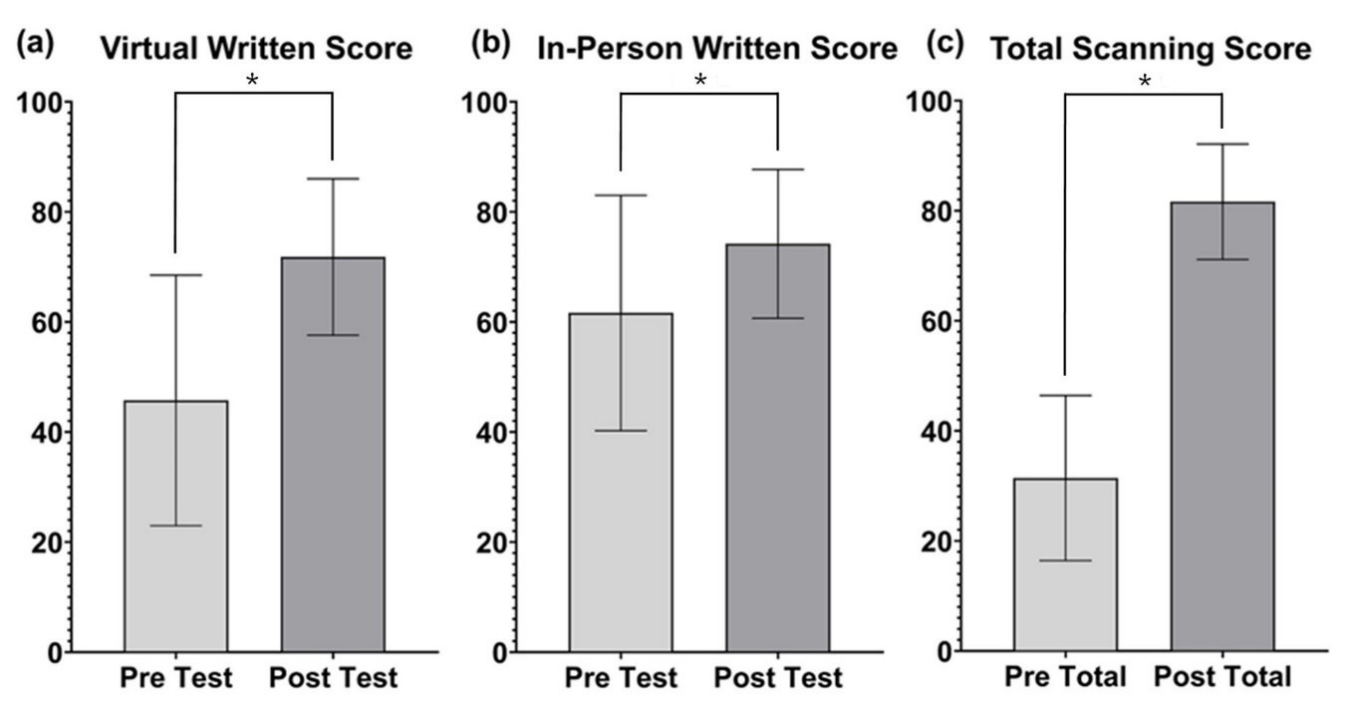
Mean and Standard Deviations of (a) Virtual Group Written Test Scores (b) In-person Group Written Test Scores and (c) In-Person Group Combined Objective Structured Assessment of Ultrasound Skills (OSAUS) Scores expressed as a percentage of Total scores before and after each learning intervention. *(P<0.001).

Student performance was assessed before and after the teaching session. In the in-person group, the mean pre-test score was 61.6 ± 21.5%, the post-test score was 74.2 ± 13.6% and mean change was 12.6 ± 4.6% (P≤0.0001). In the virtual teaching group, the mean pre-test score was 45.7 ± 23.3%, the post-test score was 71.8 ± 14.5 % and the mean change was 26.1 ± 21.5 % (P≤0.0001). Pre-test scores significantly differed between online and in-person groups (P < 0.01). Post-test scores were not very different. The change in test scores between pre- and post-test scores was significantly different (P<0.01) (Figure 2).

### OSAUS Test Results: 

To assess scanning performance, students were scored out of a total of 13 points in each scanning station. The mean pre-and post-test scores in the PLAX station were 29.5 ± 16.5% and 74.8 ± 14.8%, respectively. The mean pre- and post-test scores in the anterior lung and pleura station were 33.0 ± 18.3% and 78.5 ± 14.4%. In the RUQ abdominal station, the pre- and post-test scores were 31.7 ± 19.6% and 83.9 ± 12.9%. The mean changes were 45.5 ± 21.0%, 45.5 ± 20.5% and 52.2 ± 20.3% (all P < 0.001) for PLAX, anterior lung and pleura, and RUQ stations, respectively (Figure 2). 

### Follow-up Survey Results: 

**Figure 3:**
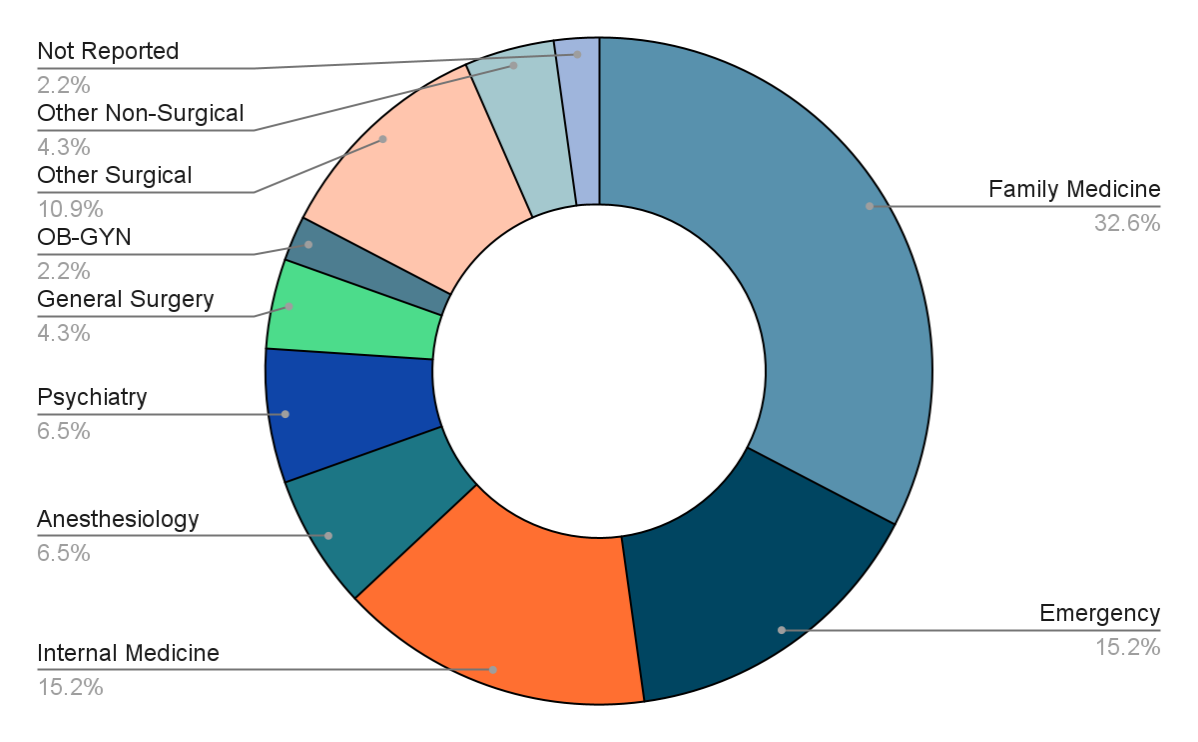
Specialty training program of the follow-up survey respondents (n=46).

Forty-six responses were received in the follow-up survey. Forty respondents completed the in-person session, and six respondents completed the virtual session. Among these, 15 respondents (32.6%) were Family Medicine trainees, 7 respondents (15.2%) were Emergency Medicine trainees, 7 respondents (15.2%) were Internal Medicine trainees, and 3 respondents (6.5%) were Anesthesiology trainees. The respondents’ speciality training programs are summarized in Figure 3. 

**Figure 4:**
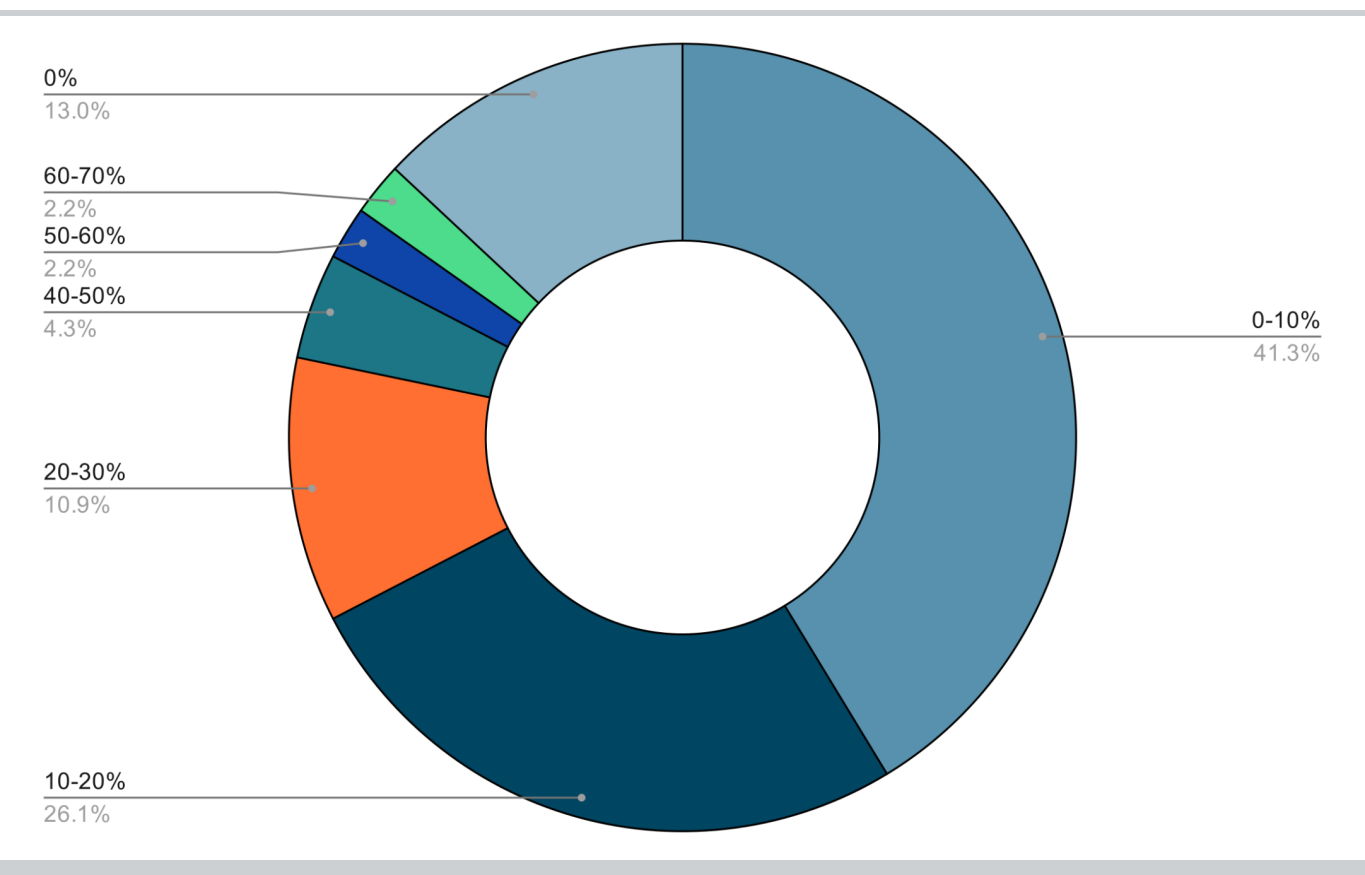
Self-reported utilization of POCUS from the follow-up survey as a percentage of patient interactions in residency (n=46). This value includes all their rotations so far and stratified into 10% ranges.

Internal Medicine, Emergency Medicine, and Anesthesiology residents reported the highest frequency of POCUS utilization, with the median estimating its use in 20-30% of clinical encounters. Respondents from other residency programs used POCUS less or not at all. The self-reported utilization of POCUS in residency is available in Figure 4. Additional self-reflecting questions about the residents' own POCUS learning are found in Table 1. 

**Table 1:** Participants’ thoughts on the training session now as residents (%) (n=46).

	Strongly Disagree	Disagree	Neither Agree nor Disagree	Agree	Strongly Agree	N/A
Q1: I am confident about my ability to apply POCUS in clinical settings	2.2%	37.0%	37.0%	23.9%	0%	0%
Q2: I have had adequate exposure to POCUS for my level of responsibility	2.2%	19.6%	19.6%	50%	8.7%	0%
Q3: This study's online POCUS module (not including hands-on training) was helpful for my application of POCUS independently	0%	8.7%	28.3%	50.0%	8.7%	4.3%
Q4: This study's in-person hands-on POCUS training was helpful to my application of POCUS independently (select N/A if you were virtual-only)	0.0%	0.0%	17.4%	58.7%	13.0%	10.9%

## Discussion

This study demonstrated that live and virtual POCUS teaching sessions could improve medical students’ theoretical POCUS knowledge. The in-person learning outcomes were consistent with a study by Heiberg et al. that showed in a 16-participant single-centre study that medical students with no prior ultrasound experience improved in image acquisition and interpretation after an online module and improved further after 4 hours of hands-on training 7. In contrast, Mackay et al. demonstrated in a small 14-participant, single-centre study that students who completed self-directed learning modules could not achieve competence in POCUS 8. The discrepancy may be attributed to the difference in teaching methodology. The difference in methods highlights the importance of live instruction for teaching POCUS. 

In this study, the difference in cohort sizes is attributed to the emergence of COVID-19 physical distancing recommendations during the study. Initially, all participants planned to attend the in-person training. This modified study design emerged because of the interruption of previous educational activities and provided the opportunity to study in-person and virtual teaching methods in tandem. Due to the ad hoc adaptation to the virtual learning platform, the virtual learning group had less time to review the pre-session resources. This likely accounted for the lower pre-test scores in the virtual group. Despite lower pre-test scores, the virtual group performed similarly to the in-person group on the post-test. 

The follow-up survey demonstrates the increasing prevalence of POCUS, with 46% of participants using POCUS in at least 10% of cases and 20% of participants using it in more than 20%, especially in Internal Medicine, Emergency Medicine, or Anesthesiology residency programs. However, this increasing prevalence of POCUS is yet to be reflected in undergraduate medical curricula. In a 2016 survey, approximately half of 13 Canadian medical schools had POCUS teaching at the undergraduate level, and of those schools 50% had an estimated 1-5 hr of teaching per year 1.

The adaptations to conventional POCUS teaching described in this study resulted directly from physical distancing recommendations during the COVID-19 pandemic. While many learners have now returned to the in-person classroom, these innovations offer distinct advantages that can be carried forward into future POCUS programs. Virtual teaching addresses commonly cited barriers to POCUS education: limitations in expertise and infrastructure 3, 4. In contrast to pre-recorded lectures, it also allows for interactivity between students and instructors. Basic demonstrations can be delivered to a group as large as 30 people, as was done in this study. The scalability of virtual demonstration sessions, requiring one probe and a simple audiovisual setup, can be an effective way to teach POCUS basics. While virtual POCUS teaching cannot fully replace conventional teaching, and students should still have hands-on experience, a hybrid and flipped-classroom teaching approach can be a useful addition to the educational toolkit.

We believe that this preliminary POCUS training model may serve as a blueprint for other institutions aiming to migrate POCUS teaching to the virtual learning environment. Employing this methodology reduces the required infrastructure and increases accessibility to POCUS training for a large group of novice users. Further studies should investigate whether this correlates with scanning proficiency. Further development of remote POCUS education should focus on the feasibility and efficacy of remote training where learners use a loaned POCUS device while remotely mentored by a POCUS expert. 

## Conclusion

This study describes an in-person POCUS teaching model that was adapted to a virtual model during the early COVID-19 pandemic. In this study, second-year medical students improved foundational knowledge scores and practical scanning performance after in-person teaching, while a virtual group showed similar improvement in knowledge scores after implementing an adapted POCUS training experience. The approach to teaching and acquisition of ultrasound skills is currently undergoing profound change. As we move beyond universal physical distancing restrictions, these innovations are lessons we can take forward as part of our teaching toolkit. Virtual POCUS teaching is scalable, and it uses minimal infrastructure and retains the interactivity of conventional small-group POCUS teaching. As an adjunct to conventional in-person POCUS teaching, these virtual teaching modalities allow educators to meet learners’ needs, regardless of physical, resource or geographical limitations. 

## Conflict of Interest Declaration

The project was funded by the Southeastern Ontario Academic Medical Organization (SEAMO) Endowed Education and Scholarship Fund. The authors report no conflict of interest. 

## Supplementary Material

Supplementary Item 1 OSAUS Assessment Form.

Supplementary Item 2 Equipment Set up and Details.
