# Supplementary material for: Lessons Learned from POCUS Instruction in Undergraduate Medicine During the COVID-19 Pandemic
Source: POCUS J. 2023 Apr 26;8(1):81–7. doi: 10.24908/pocus.v8i1.16410 (PMC10155734; doi:10.24908/pocus.v8i1.16410)
Supplement: Supplementary Item 1 [file pocusj-08-16410-s001.pdf]

POCUS Study 2020 Assessment Sheet

Student Name: ___________________________
Assessor Name: __________________________
Pre Test / Post Test (circle one)

	**Parasternal Long Axis**	**Anterior Pleura / Lung**	**Right Upper Quadrant**
**Please obtain a POCUS _________ view. You will have ~2 minutes. Verbalize your actions and notify your assessor when you have your view.**			
**Transducer Choice**<br>Correct transducer for this examination	Phased array probe<br>▢ Incorrectly Performed<br>▢ Correctly Performed	Any probe<br>▢ Incorrectly Performed<br>▢ Correctly Performed	Curvilinear / phase-array<br>▢ Incorrectly Performed<br>▢ Correctly Performed
**Probe Placement**<br>Probe was placed in the correct initial anatomical location	(left parasternal 2-5th intercostal space with indicator pointed towards the right shoulder)<br><br>▢ Incorrect Location<br>▢ Partially Correct<br>▢ Correct Location	(anterior chest, indicator to head, perpendicular to chest wall)<br><br>▢ Incorrect Location<br>▢ Partially Correct<br>▢ Correct Location	(posterior axillary line 8-11 ribs, indicator cephalad angled posteriorly slightly)<br><br>▢ Incorrect Location<br>▢ Partially Correct<br>▢ Correct Location
**Knobology**<br>Presets, depth and gain were adjusted appropriately for the image	▢ Incorrect optimization<br>▢ Some errors<br>▢ Adequate optimization	▢ Incorrect optimization<br>▢ Some errors<br>▢ Adequate optimization	▢ Incorrect optimization<br>▢ Some errors<br>▢ Adequate optimization
**Image Quality**<br>Are students generating the image asked of them	▢ No image<br>▢ Poor image<br>▢ Adequate image<br>▢ Good image<br>▢ Optimal image	▢ No image<br>▢ Poor image<br>▢ Adequate image<br>▢ Good image<br>▢ Optimal image	▢ No image<br>▢ Poor image<br>▢ Adequate image<br>▢ Good image<br>▢ Optimal image
**Please describe three structures on your view - if no view is obtained, please describe three structures on an optimal view**			
**Basic Knowledge**<br>Student was able to describe three structures visualized on their screen / what would optimally be seen	(RV, LV, LA, LVOT, aortic valve, interventricular septum)<br><br>▢ 1 or 0 structures<br>▢ 2 Structures named<br>▢ 3 Correct structures	(ribs, pleura, lung, soft tissue, rib shadows, A-lines)<br><br>▢ 1 or 0 structures<br>▢ 2 Structures named<br>▢ 3 Correct structures	(diaphragm, liver, kidney, hepatorenal space, spine)<br><br>▢ 1 or 0 structures<br>▢ 2 Structures named<br>▢ 3 Correct structures
**Gel and Hygiene**<br>Adequate gel, probe hygiene was used prior and after examination	▢ Inadequate gel, no probe hygiene<br>▢ Some errors in probe hygiene / gel<br>▢ Correct use of gel and probe hygiene		
