# Supplementary material for: Lessons Learned from POCUS Instruction in Undergraduate Medicine During the COVID-19 Pandemic
Source: POCUS J. 2023 Apr 26;8(1):81–7. doi: 10.24908/pocus.v8i1.16410 (PMC10155734; doi:10.24908/pocus.v8i1.16410)
Supplement: Supplementary Item 2 [file pocusj-08-16410-s002.pdf]

Supplementary Item 2: Equipment Set up and Details.

The equipment required for the live demonstration included a microphone, a laptop with live streaming capabilities and a video camera, a mobile device compatible POCUS device, a screen-sharing enabled video conferencing software and either a POCUS phantom to simulate anatomy or a participant willing to be scanned. We used a Philips Lumify (Amsterdam, Netherlands) device connected to a Samsung Galaxy tablet (Seoul, South Korea), however this would be capable with any mobile device compatible POCUS device. The laptop's video camera allows instructors to demonstrate external landmarking, probe placement and probe handling. The acquired POCUS image was shared from the mobile device via the video conferencing software. We trialed both Zoom (San Jose, CA, USA) and Reacts by Innovative Imaging Technologies (IIT) (Montreal, Quebec, CA) for this function. With this setup, students were able to correlate probe movements with changes in the acquired POCUS image.
